# Perforated Necrotizing Cholecystitis, Niemeier Type II, With Subcutaneous Abscess in a Frail Elderly Patient: Challenges in Diagnosis and Management

**DOI:** 10.7759/cureus.109523

**Published:** 2026-05-23

**Authors:** Michail Angelos Papaoikonomou, Nikolaos Krokos, Angeliki Chlorou, Ioannis Skandalos, Georgios Tsavdaris

**Affiliations:** 1 Department of General Surgery, Agios Pavlos General Hospital, Thessaloniki, GRC

**Keywords:** cholecystostomy, necrotizing cholecystitis, perforated cholecystitis, pseudomonas aeruginosa, subcutaneous pericholocystic abscess

## Abstract

Perforated necrotizing cholecystitis is a life-threatening complication of acute cholecystitis (AC), particularly in frail elderly patients, in whom atypical presentations may delay diagnosis. We report the case of an 89-year-old man with significant cardiovascular, cerebrovascular, and renal comorbidities who presented with abdominal pain, nausea, and a palpable right upper quadrant mass mimicking gallbladder hydrops at presentation. Laboratory findings showed leukocytosis, elevated inflammatory markers, and acute-on-chronic renal dysfunction. Contrast-enhanced CT revealed perforated necrotizing cholecystitis with a large 13 × 3.4 cm loculated pericholecystic abscess.

Given the hostile operative field and high surgical risk, the patient underwent open drainage and cholecystostomy. Cultures grew multidrug-resistant *Pseudomonas aeruginosa* sensitive only to colistin. Despite targeted antimicrobial therapy and intensive care support, the patient developed multi-organ failure and died on postoperative day eight.

This case highlights the diagnostic challenges of atypical presentations in elderly patients, emphasizing the importance of early CT imaging, prompt source control, and consideration of multidrug-resistant pathogens. Mortality remains high in the presence of advanced organ dysfunction.

## Introduction

Progressive population aging and the increasing prevalence of gallstones in older individuals have made acute cholecystitis (AC) one of the most common causes of emergency surgical admission. AC complicates 10-20% of gallstone-related admissions, and gallbladder perforation occurs in 2-15% of these patients, with mortality rates of up to 30-50%, particularly in necrotizing forms in elderly patients with comorbidities due to delayed diagnosis and sepsis [1-3]. Elderly patients are at significant risk of developing AC, with up to 6% of elderly people suffering from severe AC. Necrotizing/gangrenous cholecystitis, characterized by gallbladder ischemia and wall necrosis, represents a severe complication occurring in approximately 2-30% of AC cases and appears to be more frequent in elderly patients with multiple comorbidities. Reported rates in older populations range from approximately 18-29%, particularly among older male patients with diabetes and cardiovascular disease [4]. Laparoscopic cholecystectomy (LC) is the current gold standard for treating acute calculous cholecystitis, with early intervention preferred. However, in elderly patients, disease severity, comorbidities, and poor functional status increase the risks of surgical intervention, potentially leading to higher morbidity and mortality. Most studies define elderly patients as individuals aged ≥65 years [5]. According to the Tokyo Guidelines 2018 (TG18), severity grading of AC guides management, with early LC recommended for Grade I-II disease and gallbladder drainage advised for Grade III disease in high-risk surgical candidates [6].

Contained perforations may present without peritonitis, mimicking hydrops on physical examination. Similar cases with perforation have been associated with virulent pathogens such as *Pseudomonas aeruginosa* in patients with diabetes or renal failure and have also been reported after the use of contaminated endoscopes during endoscopic retrograde cholangiopancreatography [7]. This report describes a case of perforated necrotizing cholecystitis with a large pericholecystic abscess in a frail elderly man, emphasizing diagnostic pitfalls, surgical decision-making, and antimicrobial challenges in the era of multidrug resistance. This case report is presented in accordance with the SCARE 2023 criteria.

## Case presentation

An 89-year-old man presented to the ED with a two-day history of abdominal pain, nausea, and progressive abdominal distension. Physical examination revealed a distended abdomen without peritoneal signs, a positive Murphy’s sign, and a palpable right upper quadrant mass, clinically suggestive of a markedly distended gallbladder, similar to the appearance seen in gallbladder hydrops. The patient was transferred from a geriatric rehabilitation facility. His past medical history was significant for severe cardiovascular and cerebrovascular disease, including coronary artery disease requiring four prior coronary artery bypass graft (CABG) procedures and four documented ischemic stroke episodes with residual functional decline. He also had long-standing arterial hypertension, type 2 diabetes mellitus, benign prostatic hyperplasia, and chronic kidney disease with a baseline serum creatinine of 1.9 mg/dL. On admission, vital signs were stable, with no fever, tachycardia, or hypotension documented: blood pressure, 143/77 mmHg; oxygen saturation (SpO₂), 97%; heart rate, 80 beats/minute; and temperature, 36.8°C.

Initial laboratory evaluation demonstrated leukocytosis with marked neutrophilia, elevated inflammatory markers, impaired renal function consistent with acute-on-chronic kidney failure, and normocytic anemia. Liver biochemistry revealed elevated cholestatic enzymes without significant hyperbilirubinemia. Laboratory findings are summarized in Table 1.

**Table 1:** Laboratory findings on admission. Hb: Hemoglobin; Hct: Hematocrit; PLT: Platelet count; AST: Aspartate aminotransferase; ALT: Alanine aminotransferase.

Parameter	Value	Units	Reference range
Complete blood count			
WBC count	12.3	× 10³/µL	4.2-11
Neutrophils	90.3	%	40-70
Lymphocytes	6.2	%	20-45
Hemoglobin (Hb)	9.2	g/dL	14-17.4
Hematocrit (Hct)	29.2	%	42-52
Platelet count (PLT)	146	× 10³/µL	140-390
Biochemistry			
Glucose	250	mg/dL	70-109
Urea	170	mg/dL	17-52
Creatinine	2.41	mg/dL	0.72-1.25
Potassium	3.5	mmol/L	3.5-5.1
Sodium	144	mmol/L	136-145
Total bilirubin	0.49	mg/dL	0.20-1.20
Direct bilirubin	0.31	mg/dL	0.10-0.50
AST	30	IU/L	5-34
ALT	10	IU/L	<55
Gamma-GT	41	IU/L	12-64
Alkaline phosphatase	300	IU/L	40-150
Amylase	61	IU/L	25-125
Lipase	60	U/L	8-78
C-reactive protein (CRP)	10.82	mg/dL	<0.5

No definite pneumoperitoneum was seen on supine abdominal radiograph (Figure 1A). Initial abdominal USG demonstrated a markedly distended gallbladder measuring approximately 13.6 × 4.1 cm, containing dense echogenic biliary sludge without clear posterior acoustic shadowing. The gallbladder wall appeared focally thickened, with heterogeneous hypoechoic areas within the wall suggestive of intramural microabscess formation. No definite sonographic evidence of free intraperitoneal fluid was observed (Figures 1B-1C).

**Figure 1:**
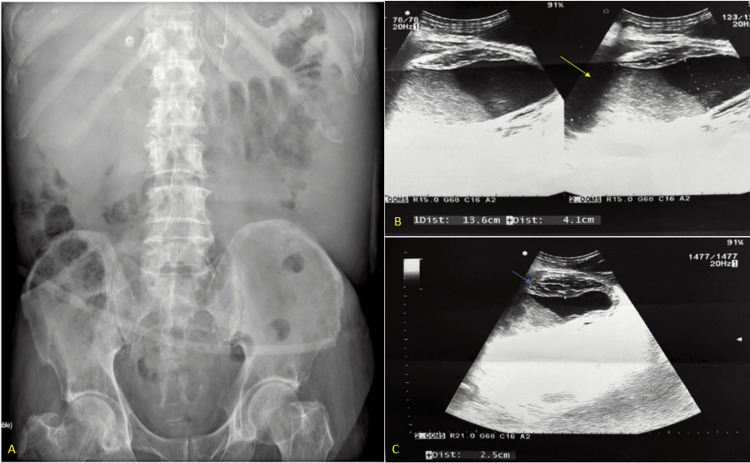
Imaging investigation of the case. (A) Abdominal X-ray showing no definite pneumoperitoneum or other evident acute abdominal pathology. (B, C) Preoperative abdominal USG. (B) Longitudinal view demonstrating a markedly distended gallbladder measuring 13.6 × 4.1 cm, with internal echogenic biliary sludge (yellow arrow). (C) Focal gallbladder wall thickening measuring approximately 2.5 cm, with heterogeneous hypoechoic intramural areas suggestive of microabscess formation (blue arrow).

These findings were suspicious for complicated AC, prompting further evaluation with contrast-enhanced computed tomography. Abdominal CT demonstrated a large right upper-quadrant loculated subcutaneous fluid collection measuring approximately 13 × 3.4 cm, associated with marked pericholecystic inflammatory fat stranding. The findings were consistent with contained perforation of acute necrotizing cholecystitis and pericholecystic abscess formation, as shown in Figure 2.

**Figure 2:**
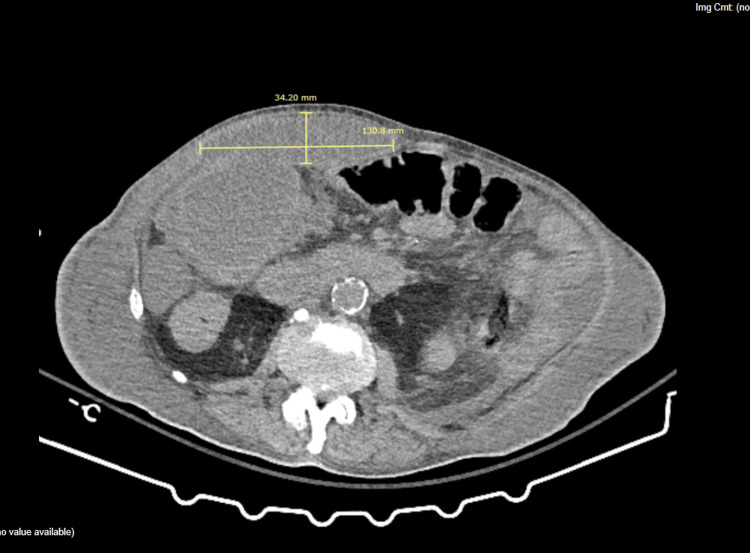
Abdominal CT scan depicting a large pericholecystic abscess due to perforated necrotizing cholecystitis.

Following laboratory and imaging findings, the patient was taken urgently to the operating room. A right subcostal incision was made. Intraoperatively, a large inflammatory collection was encountered and drained, and microbiological cultures were obtained. Upon entry into the abdomen, thick purulent material mixed with necrotic debris was visualized, confirming advanced suppurative/necrotizing infection. The yellow-green, friable tissue corresponded to necrotic gallbladder wall and inflamed pericholecystic fat, reflecting ongoing ischemia and tissue breakdown (Figures 3A-3B).

**Figure 3:**
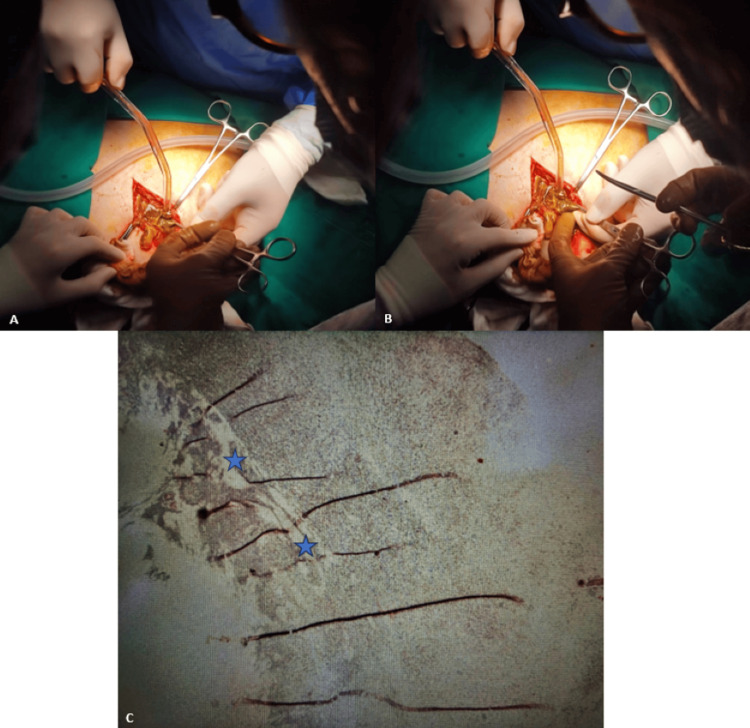
Intraoperative and histopathologic findings. (A, B) Intraoperative findings during open cholecystostomy and abscess drainage. The images show the operative field following a Kocher incision in a patient with perforated necrotizing cholecystitis. A localized abdominal wall opening exposes a large pericholecystic abscess cavity, consistent with contained gallbladder perforation (Niemeier type II). (C) Histopathologic image of the surgical specimen showing part of the gallbladder fundus with necrosis and abscess formation within the wall (blue asterisks) at 10× magnification; H&E stain.

The operative field appeared markedly inflamed and edematous, illustrating the hostile surgical environment described in the case, which precluded safe definitive cholecystectomy. The hostile operative field justified the decision to proceed with damage-control surgery using open drainage and cholecystostomy with Pezzer tube placement. The removed portion of the gallbladder fundus was sent for histological examination. The pathology report described necrotic foci and abscess formation of the fundus wall associated with acute severe cholecystitis, with no evidence of coexisting neoplasia (Figure 3C).

Empiric broad-spectrum antimicrobial therapy was initially initiated with a tigecycline loading dose of 100 mg, followed by 50 mg, in combination with amikacin 500 mg every 12 hours. Microbiological cultures obtained from the intra-abdominal fluid subsequently grew *Pseudomonas aeruginosa*, demonstrating susceptibility only to colistin. Antimicrobial therapy was therefore escalated to include colistin, 3 million units every 12 hours, together with meropenem-vaborbactam 2 g every 8 hours. Despite aggressive antimicrobial and supportive treatment, the patient developed progressive multi-organ deterioration dominated by renal failure requiring hemodialysis via temporary vascular access and ultimately died during hospitalization after a total hospital stay of eight days, including seven days in the ICU.

## Discussion

Acute cholecystitis is the most prevalent complication of cholelithiasis and one of the primary causes of emergency surgery in elderly patients. Cholelithiasis causes 90-95% of all AC cases, with acalculous cholecystitis accounting for the remaining 5%-10%. Up to 20-40% of asymptomatic people with gallstones will eventually develop symptoms, with an annual incidence of 1-3%, and in 10-15% of patients with AC, AC is the first symptomatic manifestation of gallstone disease [8]. Perforated necrotizing cholecystitis represents one of the most severe forms of gallbladder inflammation and remains associated with high morbidity and mortality, particularly in elderly patients with multiple comorbidities. Preoperative diagnosis has been reported in only 3% of cases [9]. While gallbladder perforation occurs in a minority of AC cases, approximately 2-15%, its clinical significance lies in its frequently atypical presentation and rapid progression to sepsis, both of which were evident in this case [1-3].

Necrotizing cholecystitis results from sustained cystic duct obstruction leading to ischemia, transmural necrosis, and eventual perforation. Elderly patients with diabetes and vascular disease are particularly vulnerable because of microangiopathy, impaired immune response, and delayed symptom perception [10]. Niemeier proposed the classification of gallbladder perforation, which Anderson BB and Nazem A subsequently modified in 1987 [11]. This classification remains the most extensively used for gallbladder perforation. It identifies three primary clinical subtypes, while Anderson BB and Nazem A proposed a fourth category, as shown in Table 2.

**Table 2:** Niemeier classification of gallbladder perforation, including the Anderson modification. Adapted from reference [11].

Type	Description	Clinical features	Prognosis / notes
Type I	Acute free perforation into the peritoneal cavity	Generalized peritonitis, acute abdomen, sepsis	Most severe form; requires emergency surgery; associated with high mortality
Type II	Subacute perforation with localized pericholecystic abscess	Localized right upper quadrant pain, palpable mass, and possible absence of generalized peritonitis	Most common type; often seen in elderly patients; may mimic gallbladder hydrops
Type III	Chronic perforation with internal fistula formation, usually cholecystoenteric fistula	Cholecystoenteric fistula; possible gallstone ileus	Chronic course; may present with bowel obstruction
Type IV - Anderson modification	Chronic perforation with cholecystobiliary fistula, involving communication between the gallbladder and biliary tree	Atypical biliary symptoms, jaundice, recurrent cholangitis, or incidental detection	Rare; represents internal biliary fistulization rather than enteric communication

According to the Niemeier classification, this case corresponds to Type II perforation, defined as subacute perforation with localized abscess formation, which is the most commonly encountered form in elderly patients [11]. Although contained, Niemeier type II, perforations are the most common subtype in elderly patients, they typically present with localized pain and/or sepsis.

In contrast, our patient demonstrated a minimal systemic inflammatory response and a palpable mass mimicking gallbladder hydrops, highlighting the well-documented but under-recognized phenomenon of blunted inflammatory response in frail, elderly, and diabetic individuals [12]. This atypical presentation contributed to delayed recognition and reflects a growing diagnostic challenge in aging populations. Recent studies further emphasize that advanced age and comorbidities significantly influence disease progression and clinical severity [13]. A 2023 study showed that perforated cholecystitis has a higher prevalence in elderly male patients with multiple comorbidities, especially diabetes mellitus, hypertension, and dyslipidemia [2]. This case is consistent with these findings, as the presence of extensive cardiovascular disease, recurrent cerebrovascular events, and chronic renal impairment significantly increased operative risk and contributed to the unfavorable outcome. The vast majority of patients diagnosed with this condition are obese or have diabetes [2]. Obesity might increase the risk of gallbladder perforation because of its associated comorbidities, whereas diabetes can cause neuropathy, masking characteristic symptoms until perforation occurs and, therefore, causing a delay in diagnosis [10]. Furthermore, diabetes may decrease WBC activity, resulting in immunosuppression [2]. Although major necrosis and perforation developed in our case, the severity of the symptoms was unexpected, and the clinical findings at the facility were evaluated seriously only after nausea and vomiting began. Gallbladder perforation necessitates immediate care, including surgical intervention and drainage, antimicrobial coverage, and fluid resuscitation [14].

According to the literature, the risk of gangrenous cholecystitis increases in patients over the age of 50, with admission factors including temperature >37.2°C, heart rate >90 beats per minute, and WBC >14 × 10³/μL. Other identified factors, such as gallbladder wall thickening >4 mm, serum sodium ≤135 mmol/L, and hyperbilirubinemia, have also been reported as statistically relevant in the development of this form of cholecystitis [15]. Imaging plays a pivotal role in such scenarios [16]. While USG remains the first-line modality, it may underestimate disease severity in complicated cases. In cases of acute perforated cholecystitis, registry data demonstrate that CT is employed more than twice as often as in non-perforated cholecystitis [17]. In line with current evidence, this case reinforces the importance of early contrast-enhanced CT, which remains the most sensitive modality for detecting perforation, abscess formation, and the extent of inflammatory spread. Contemporary literature strongly supports early CT utilization in elderly patients with atypical abdominal findings, as delays are directly associated with worse outcomes and high mortality [16]. In addition, contrast-enhanced CT is pivotal in delineating loculated collections and fat stranding to guide intervention [2,16].

The Tokyo Guidelines provide standardized criteria for diagnosing AC, grading its severity, and guiding the timing and type of intervention, including medical management, drainage, or surgery [14,18]. Based on TG18 criteria, the presence of renal dysfunction, acute-on-chronic renal failure, categorizes this case as Grade III, severe, AC [6]. TG18 defines renal dysfunction as serum creatinine >2.0 mg/dL as one of the criteria for Grade III, severe, acute cholangitis/cholecystitis-associated organ dysfunction. However, because the patient had pre-existing chronic kidney disease with an already elevated baseline creatinine, distinguishing true acute organ dysfunction from chronic impairment or mild acute-on-chronic deterioration is challenging. According to the Tokyo Guidelines 2018, gallbladder drainage is recommended for Grade III cholecystitis in high-risk patients (Table 3).

**Table 3:** Severity grading and management of acute cholecystitis according to the Tokyo Guidelines 2018. Adapted from references [14,18]. AbbreviationFull form ASA-PS: American Society of Anesthesiologists Physical Status; RUQ: Right upper quadrant.

Severity grade	Clinical / laboratory / imaging features	Organ dysfunction / comorbidities	Recommended timing and type of intervention
Grade I (mild)	Acute cholecystitis that does not meet the criteria for Grade II or Grade III disease. Clinical features may include right upper quadrant (RUQ) pain/tenderness and Murphy’s sign, with systemic inflammatory findings such as fever, leukocytosis, elevated CRP, and imaging-confirmed acute cholecystitis.	No organ dysfunction; low surgical risk, usually with limited or mild comorbidities, such as American Society of Anesthesiologists physical status (ASA-PS) ≤2.	Early laparoscopic cholecystectomy when anatomical and technical conditions allow and the patient is fit for surgery.
Grade II (moderate)	Acute cholecystitis with one or more moderate severity features, such as marked leukocytosis, palpable tender right upper quadrant mass, symptom duration >72 hours, or marked local inflammation, including gangrenous cholecystitis, pericholecystic abscess, hepatic abscess, biliary peritonitis, or emphysematous cholecystitis.	No Grade III organ dysfunction; comorbidities may increase operative risk.	Early laparoscopic cholecystectomy by experienced surgeons if the patient’s risk profile is acceptable; otherwise, initial medical treatment with or without gallbladder drainage, followed by delayed cholecystectomy once stable.
Grade III (severe)	Acute cholecystitis associated with systemic severity due to organ dysfunction. Imaging may show acute cholecystitis with complications such as perforation, abscess, or gangrenous changes, depending on the case.	At least one organ/system dysfunction, including cardiovascular, neurologic/CNS, respiratory, renal, hepatic, or hematologic dysfunction. Severe comorbidities may further increase operative risk.	First-line management usually includes intensive medical treatment with or without gallbladder drainage. Cholecystectomy is generally deferred until organ function stabilizes. Early laparoscopic cholecystectomy may be considered only in highly selected patients at experienced centers under strict criteria.

However, the presence of a large, loculated abscess and a hostile operative field made percutaneous drainage suboptimal, necessitating open surgical drainage with cholecystostomy. Furthermore, the presence of friable tissue and a hostile inflammatory environment suggested the need for direct evacuation of purulent material, debridement of necrotic tissue, and assessment of the perforated gallbladder wall. Therefore, open drainage with cholecystostomy was selected as a damage-control strategy to achieve more reliable source control in a frail, high-risk patient. Management decisions in this patient highlight an important divergence between guideline recommendations and real-world surgical practice. This underscores a key clinical insight: while minimally invasive approaches are preferred, open damage-control surgery remains indispensable in selected complex cases, particularly when adequate source control cannot be achieved otherwise. In challenging operative fields, exacerbated by adhesions and inflammation, cholecystostomy offers a temporizing drainage option, with reported success rates of 70-85% in high-risk cohorts, followed by interval cholecystectomy when feasible [19].

Perforated cholecystitis is associated with significantly worse outcomes compared with uncomplicated disease, including higher rates of conversion to open surgery, bile duct injury, re-intervention, mortality, and prolonged hospitalization [17]. Furthermore, the choice of an open approach over a laparoscopic approach may be considered appropriate in selected complex cases, as LC has been associated with a greater need for reoperations despite a shorter hospital stay in some reports (median 15 vs. 5 days) [20]. In the present case, the decision to perform open drainage with cholecystostomy was driven by patient-specific factors rather than a preference for open surgery over laparoscopic surgery. The patient was an 89-year-old frail individual with multiple comorbidities, perforated necrotizing cholecystitis, a large loculated abscess, and a markedly hostile operative field with extensive inflammation and tissue necrosis. Under these circumstances, definitive cholecystectomy was considered technically hazardous and associated with substantial operative risk. Therefore, the primary objective was prompt and adequate source control through a damage-control strategy consisting of abscess drainage and cholecystostomy. This approach reflects individualized surgical decision-making in a high-risk patient rather than evidence supporting the general superiority of open surgery. While LC remains the preferred approach in appropriately selected patients, management in complex cases must be tailored according to physiologic reserve, local disease extent, and intraoperative findings. Early intervention is also considered appropriate in these cases, as it leads to fewer open surgeries, fewer surgical site infections, and a shorter overall hospital stay [20].

Percutaneous cholecystostomy is a commonly used procedure to treat AC in patients with significant comorbid disease. Specifically, patients over 65 years of age who have American Society of Anesthesiologists (ASA) scores of III or IV, septic shock, or are classified as unfit for surgery may be candidates for percutaneous cholecystostomy, either ultrasound- or CT-guided [5,12,19]. In emergency situations, this procedure can be life-saving for individuals who cannot withstand immediate general anesthesia and surgery, and it can also serve as a bridge to surgery [5,12]. After thorough examination of the biliary duct using cholangiography, the cholecystostomy catheter may be removed four to six weeks after placement [5,12]. Some publications suggest watchful waiting for symptomatic cholelithiasis in patients aged >90 years and performing cholecystectomy only if the condition progresses to AC [21]. Generally, early LC is well suited only to elderly patients with mild or moderate AC [2,19].

A further distinguishing feature of this case is the isolation of multidrug-resistant *Pseudomonas aeruginosa*, an uncommon but increasingly reported pathogen in biliary infections, particularly among institutionalized and immunocompromised patients, with reported rates of approximately 1-5%. Recent epidemiological data indicate a rising incidence of healthcare-associated biliary infections caused by resistant Gram-negative organisms, which are associated with higher rates of treatment failure and mortality [7]. The need to escalate to colistin-based therapy reflects the growing global challenge of antimicrobial resistance and highlights the importance of early microbiological sampling and tailored therapy. Susceptibility-guided agents such as colistin may be helpful in such cases, as broad-spectrum empiric regimens, such as tigecycline-amikacin, may fail. Key lessons include the need for prompt CT in elderly diabetic patients with abdominal distension and vigilance for resistant pathogens.

Despite appropriate surgical and antimicrobial management, the patient developed multi-organ failure and succumbed to the disease. Perforated cholecystitis is associated with substantially worse outcomes than uncomplicated disease, particularly in elderly patients with significant comorbidities and organ dysfunction. Mortality appears to increase considerably in the presence of sepsis and multi-organ failure; however, reported rates vary widely depending on patient characteristics, disease severity, and study population. This outcome aligns with contemporary data demonstrating that mortality in perforated cholecystitis increases dramatically in the presence of organ dysfunction, often exceeding 40-60% [14]. Key adverse prognostic factors, including advanced age, renal failure, delayed diagnosis, and resistant pathogens, were all present in this case.

In summary, the novelty of this case lies in the coexistence of a hydrops-like clinical presentation without peritoneal signs, the presence of a large pericholecystic abscess forming a subcutaneous mass due to contained perforation, and infection with multidrug-resistant *Pseudomonas aeruginosa* requiring last-line antimicrobial therapy. The limitations of this single case study include the lack of autopsy confirmation and the absence of interval cholecystectomy because of the patient’s outcome.

## Conclusions

Perforated necrotizing cholecystitis in frail elderly patients may present insidiously and without classic peritoneal signs. Early CT imaging, especially in elderly and/or diabetic patients, is critical for diagnosis. In high-risk surgical candidates, cholecystostomy provides a reasonable damage-control strategy; however, mortality remains substantial when the condition is complicated by multi-organ failure and multidrug-resistant pathogens. Empiric antibiotic coverage for multidrug-resistant microorganisms should be considered in institutionalized elderly patients, guided by local antimicrobial resistance patterns and patient-specific risk factors. Multidisciplinary management and individualized risk stratification are essential in this vulnerable population.
